# A Human Multi-Epitope Recombinant Vaccinia Virus as a Universal T Cell Vaccine Candidate against Influenza Virus

**DOI:** 10.1371/journal.pone.0025938

**Published:** 2011-10-05

**Authors:** Alan G. Goodman, Paul P. Heinen, Susana Guerra, Aneesh Vijayan, Carlos Oscar S. Sorzano, Carmen E. Gomez, Mariano Esteban

**Affiliations:** 1 Department of Cellular and Molecular Biology, Centro Nacional de Biotecnología, Consejo Superior de Investigaciones Científicas, Madrid, Spain; 2 Department of Preventative Medicine and Public Health, Universidad Autónoma, Madrid, Spain; 3 Computing Unit, Centro Nacional de Biotecnología, Consejo Superior de Investigaciones Cientificas, Madrid, Spain; Instituto Butantan, Brazil

## Abstract

There is a need to develop a universal vaccine against influenza virus infection to avoid developing new formulations of a seasonal vaccine each year. Many of the vaccine strategies for a universal vaccine target strain-conserved influenza virus proteins, such as the matrix, polymerase, and nucleoproteins, rather than the surface hemagglutinin and neuraminidase proteins. In addition, non-disease-causing viral vectors are a popular choice as a delivery system for the influenza virus antigens. As a proof-of-concept, we have designed a novel influenza virus immunogen based on the NP backbone containing human T cell epitopes for M1, NS1, NP, PB1 and PA proteins (referred as NPmix) as well as a construct containing the conserved regions of influenza virus neuraminidase (N-terminal) and hemagglutinin (C-terminal) (referred as NA-HA). DNA vectors and vaccinia virus recombinants expressing NPmix (WR-NP) or both NPmix plus NA-HA (WR-flu) in the cytosol were tested in a heterologous DNA-prime/vaccinia virus-boost vaccine regimen in mice. We observed an increase in the number of influenza virus-specific IFNγ-secreting splenocytes, composed of populations marked by CD4^+^ and CD8^+^ T cells producing IFNγ or TNFα. Upon challenge with influenza virus, the vaccinated mice exhibited decreased viral load in the lungs and a delay in mortality. These findings suggest that DNA prime/poxvirus boost with human multi-epitope recombinant influenza virus proteins is a valid approach for a general T-cell vaccine to protect against influenza virus infection.

## Introduction

Millions of people worldwide are infected with influenza virus every year. Although most yearly outbreaks are characterized by fewer than 40,000 deaths in the United States, highly virulent strains can evolve that cause worldwide pandemics, resulting in a dramatically increased incidence of death. While ribavirin and oseltamvir can be used to combat infection, there has been recent emergence of strains resistant to these drugs [1], demonstrating the need for better therapeutics or vaccine strategies against influenza virus infection.

The major cause of influenza pandemics involves the combination of the two major glycoproteins on the virion surface [2]. These two glycoproteins, hemagglutinin (HA) and neuraminidase (NA), contribute to the considerable antigenic variation of influenza virus because they have 16 and 9 subtypes, respectively. When a new glycoprotein subtype appears on the virion surface, the population is immunogenically naïve to this new strain, raising the possibility of a pandemic. The worst influenza pandemic to date occurred in 1918, by the so-called “Spanish flu,” an H1N1 virus, which led to over 40 million deaths worldwide [3]. In 2005, there were outbreaks of an H5N1 virus in Southeast Asia and Europe. While this strain caused death in humans, it has been unable to be transmitted from person to person [4].

Besides increased mortality and morbidity, how else would an influenza pandemic impact society? Compounded with the fact that influenza-associated hospitalizations have increased substantially over the last two decades [5], the economic loss due to another pandemic would be unimaginable. Based on a model which takes into account variables such as different types of vaccination strategies and illness percentages, Thompson et al. proposed that another pandemic would cause up to 207,000 and 734,000 hospitalizations, and 18 to 42 million outpatient visits in the United States alone. Based upon these numbers, they estimate that due to loss of life and medical care, the economic cost of a pandemic to the United States would be up to $167 billion dollars [6].

The current vaccination strategy against influenza virus consists of a live-attenuated or killed virus vaccine regimen containing the three strains of virus (two A subtypes and one B subtype) that are thought to be most prevalent in the upcoming influenza season. Determining which strains the vaccine will contain is based on bioinformatics analysis of epidemiological data from the previous season. Since the current vaccine strategy requires changing of the vaccine formulation every year, there is a push to develop a universal vaccine [7]. Development of these next generation influenza vaccines is based on technologies utilizing recombinant proteins, virus-like particles, viral vectors, and DNA-based vaccines [8]. Another strategy is prime-boost, using DNA for priming and virus vectors expressing antigens of interest as a boost [9]. Attenuated poxvirus vectors, such as MVA and NYVAC, have been used successfully to induce a greater immune response towards HIV antigens [10], [11], [12]. In fact, one of the more successful HIV vaccine trials in humans to date utilized a poxvirus vector [13], and there are ongoing phase I/II clinical trials using the MVA poxvirus vector [12], [14], [15], [16].

Regarding influenza virus, many vaccination strategies have used the nucleoprotein (NP) as an antigen to induce immune responses since it is well-conserved across influenza virus subtypes [17], [18], [19]. However, in some cases, vaccines developed around NP have failed to provide protection [20], [21]. Recently, MVA vectors expressing influenza virus antigens have been shown to provide protection against virus challenge, even in human clinical trials [22], [23], [24]. Novel design strategies using viral antigens in combination with NP may be able to improve immunogenicity to influenza virus and provide more universal protection.

In this study, we designed recombinant influenza virus antigens for use in a DNA prime/vaccinia virus boost vaccination strategy, and studied the ability of these proteins to induce an adaptive immune response and protective response to heterologous challenge. One immunogen was designed around influenza virus NP, which included human epitopes from the M1 (matrix), NS1 (non-structural), PB1 (basic polymerase), and PA (acidic polymerase) viral proteins. The vaccinia virus construct containing NPmix was referred to as WR-NP. The other immunogen contained conserved sequences from H5N1 viruses: the N-terminal NA fused to the C-terminal HA and was combined with the NPmix to generate the viral vector WR-flu. We also generated plasmid DNA vectors from pCIneo expressing independently NPmix and HA-NA. We show that our recombinant vaccinia virus constructs grow well in cell cultures and produce the recombinant products (NPmix or both NPmix and NA-HA) in the cytoplasm of infected cells, similarly as for the DNA vectors. After the DNA prime/poxvirus boost vaccination protocol in mice we observed increased IFNγ-secreting cells, along with an increased CD4^+^ and CD8^+^ T cell response with regard to IFNγ^+^ and IFNγ^+^TNFα^+^ cells. Upon challenge with influenza virus, lung viral titers were decreased in animals vaccinated with the viral vectors expressing recombinant influenza virus proteins. Taken together, this study demonstrates how a T cell vaccine based on a DNA prime/poxvirus boost strategy containing multiple human influenza virus epitopes can reduce viral load during heterologous challenge and provides a rational design for the generation of universal influenza vaccines.

## Results

### Immunogen design and characterization of vaccinia virus vectors expressing influenza virus antigens

**Figure 1:**
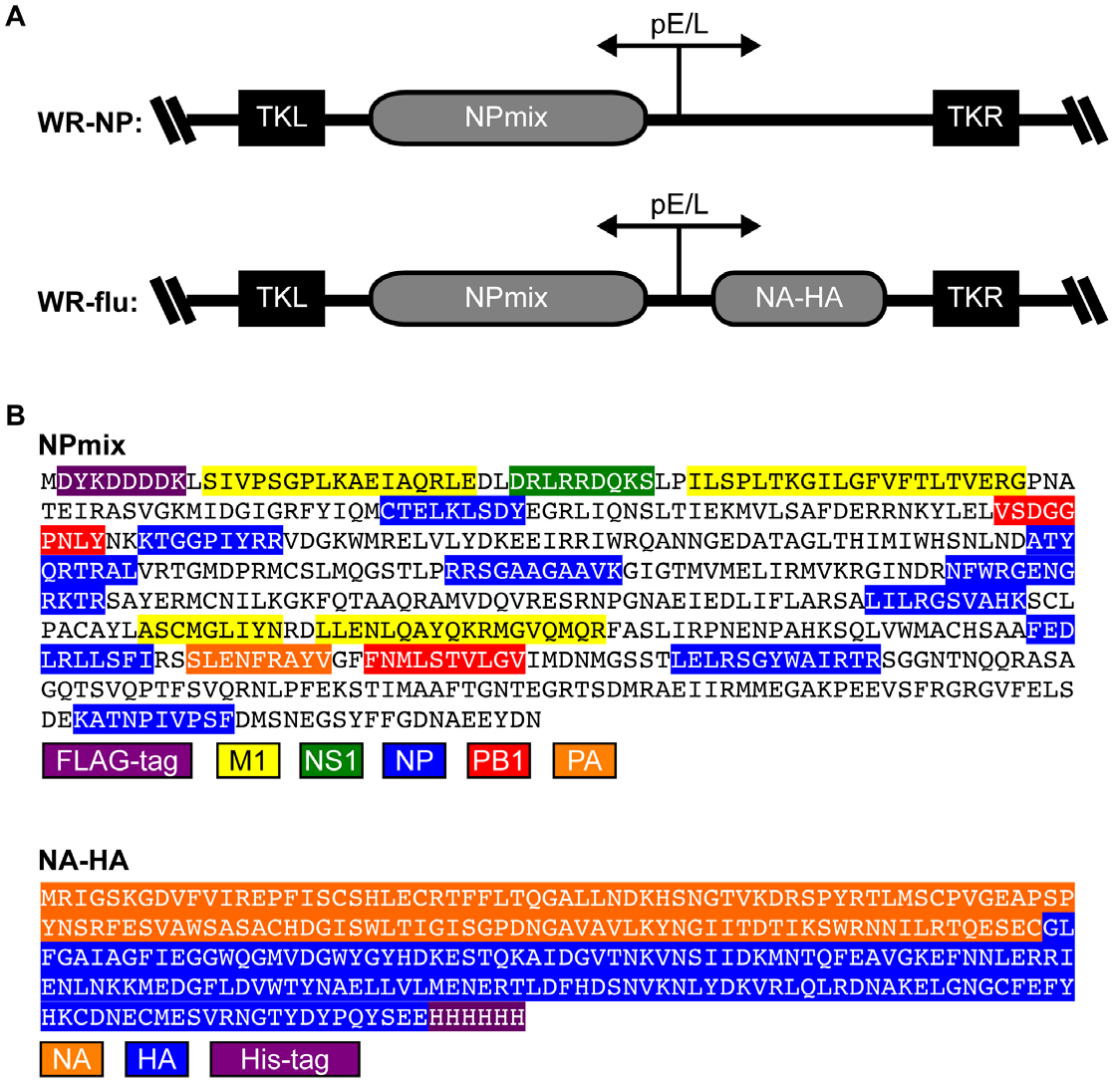
Design and characterization of vaccine constructs. (A) Recombinant influenza virus gene constructs (NPmix or NA-HA) were inserted at the TK locus of the Western Reserve (WR) of vaccinia virus and are driven by the synthetic early/late promoter (pE/L). The cloning vectors (shown) were introduced into the wild-type WR virus by homologous recombination and iterative plaque purification. (B) Amino acid sequences of NPmix and NA-HA recombinant influenza virus protein constructs. The backbone for the NPmix construct is influenza virus NP into which was inserted other influenza virus protein human T cell epitopes. Human T cell epitopes for influenza virus M1, NS1, NP, PB1, and PA proteins are indicated. The NA-HA construct consists of the conserved regions of H5N1 influenza virus neuraminidase (N-terminal amino acids 108–231) and hemagglutinin (C-terminal amino acids 347–511).

The goal in developing our T cell vaccine against influenza virus was first to design an immunogen utilizing the conserved regions of influenza virus proteins as antigens and then to produce a recombinant vaccinia virus that can be used as a broad-spectrum vaccine to induce specific immune response to influenza. For this aim, we generated two types of immunogens. One was based on the nucleoprotein (NP) as a backbone, which is well-conserved among H1N1, H2N3, H5N1, H9N2, and H7N7 strains of influenza virus (Figure S1). Within the NP backbone, we added conserved human T cell epitopes for other influenza virus proteins, specifically M1, NS1, PB1, and PA (Figure 1B; NPmix). Briefly, we were guided by Epstein, et al., which lists the influenza virus gene products and peptide sequences that are presented by certain MHC molecules [25]. We narrowed down this list to four M1, one NS1, one PA, and two PB1 epitopes that were the most conserved among the virus strains listed in Figure S1. These, in addition to the NP epitopes, were included in the NPmix recombinant gene shown in Figure 1B. In choosing the amino acids to swap, we were careful in selecting regions without significant secondary structure, and we avoided swapping hydrophilic for hydrophobic regions. We also did not remove any known NP T cell epitopes. The second construct involved fusing the conserved regions of neuraminidase (NA) and hemagglutinin (HA) from H5N1 influenza viruses (Figure 1B and S2; NA-HA). These HA and NA subtypes are not homologous those in the H3N2, H9N2, or H7N7 strains listed in Figure S1; however, the NP protein shown in Fig. S1 is conserved in the H5N1 strains. The NPmix construct alone or together with the NA-HA construct was inserted in the TK locus of the Western Reserve strain of vaccina virus (Figure 1A). We termed the vaccinia virus recombinants containing NPmix insertion alone “WR-NP” and the insertion of NPmix and NA-HA “WR-flu.”

**Figure 2:**
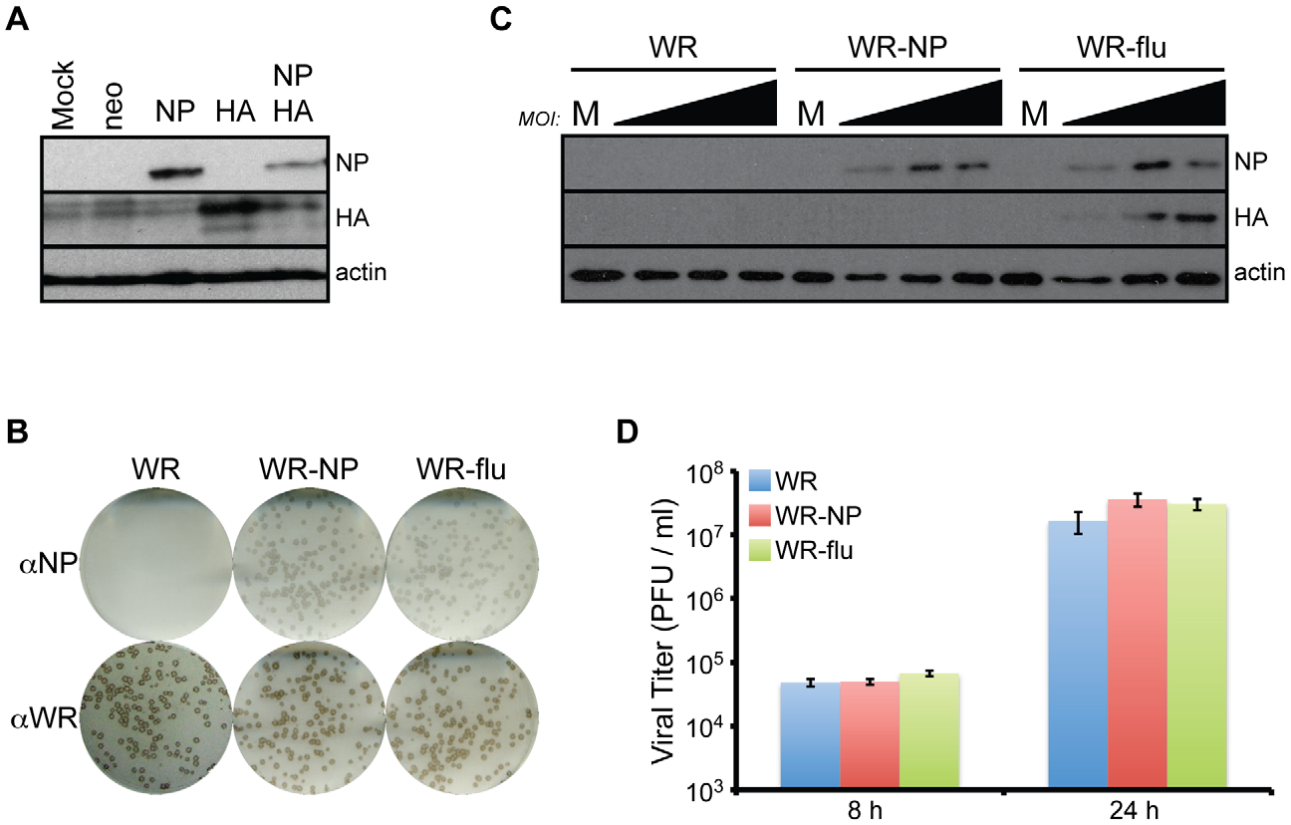
Recombinant viruses synthesize NP and HA proteins and replicate to similar levels. (A) 10 µg of pCIneo (neo) vectors containing the NPmix (NP) or NA-HA (HA) inserts were transfected into BSC40 cells. For double transfections, 5 µg of each vector was used. 48 h post-transfection, cells were lysed and levels of influenza virus proteins were determined using antibodies for NP and HA. (B–D) BSC40 cells were infected with WR, WR-NP, or WR-flu at an MOI of 0.01 (B, D), 0.1, 1, or 10 (C) PFU/cell. (B) At 24 h p.i., cells were fixed and plaques were stained with NP antibody. (C) At 24 h p.i., the levels of NP and HA in the lysates were determined by immunoblot analysis. (D) At 8 or 24 h p.i., infectious virus present in the cells was measured in triplicate standard plaque assay on BSC40 cells.

We also produced plasmid vectors for their use in the priming stages of the vaccination protocol. The NPmix and NA-HA immunogens were inserted into the pCIneo mammalian expression vector, which could efficiently express the proteins upon transient transfection in cell culture, either transfected alone or in tandem (Figure 2A). Lower levels of NP and HA were observed during double transfections since only 5 µg of each vector was used, as opposed to 10 µg in single transfections. We also verified that the influenza insert was maintained in the recombinant viruses, as shown after staining viral plaques from purified viral stocks for NP and WR protein and observing that the recombinant viruses produced the same number of plaques expressing NP and WR proteins (Figure 2B); the H5 HA antibody did not strongly stain viral plaques. We further tested how the expression of influenza virus proteins from the recombinant viruses was dependent on MOI. We observed that the NA-HA and NPmix protein is strongly expressed by 24 h p.i. during both WR-NP and WR-flu infection (Figure 2C). Finally, all viruses replicate to similar levels (Figure 2D). Together, these results indicate that the recombinant viruses are correctly expressing the influenza virus proteins and that the expression of these proteins does not alter the infection profile as compared to wild-type vaccinia virus.

**Figure 3:**
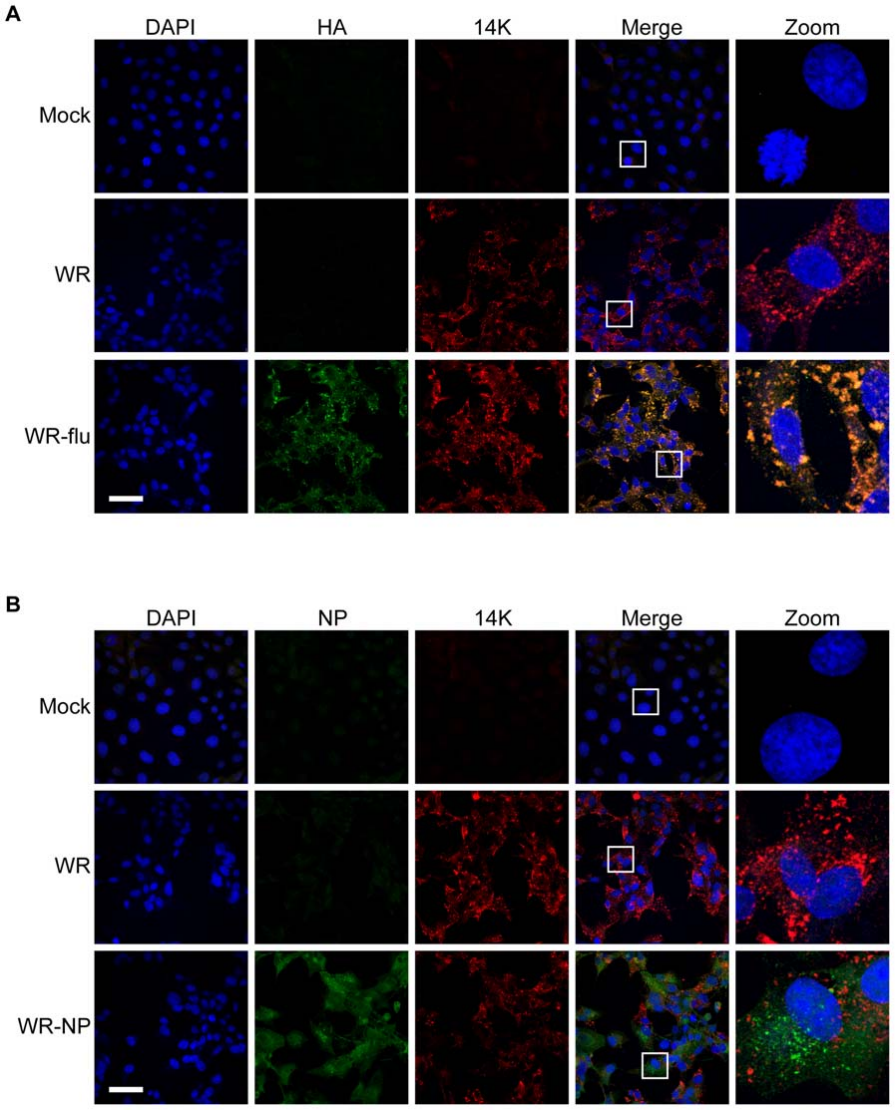
Recombinant influenza virus proteins are present in the cytosol of infected cells. BSC40 cells were infected with WR, WR-flu (A), or WR-NP (B) at an MOI of 1 PFU/cell. At 24 h p.i., cells were fixed with 2% paraformaldehyde, permeabilized, and stained with DAPI or antibodies recognizing influenza virus HA (A), NP (B), or vaccinia virus 14 K (A27 gene). Bar = 25 µm.

To further characterize the viruses, we examined the subcellular localization of the influenza virus proteins. The recombinant proteins were expressed mainly in the cytoplasm of cells during either WR-NP or WR-flu infection. HA showed a higher degree of co-localization with viral factories (Figure 3A), as marked by staining for the 14K vaccina virus protein (A27 gene), as compared to colocalization of the NP protein with 14K (Figure 3B). NPmix also exhibits greater distribution throughout the cell.

### Immunogenicity of vaccinia virus recombinants during DNA prime/poxvirus boost vaccination

**Figure 4:**
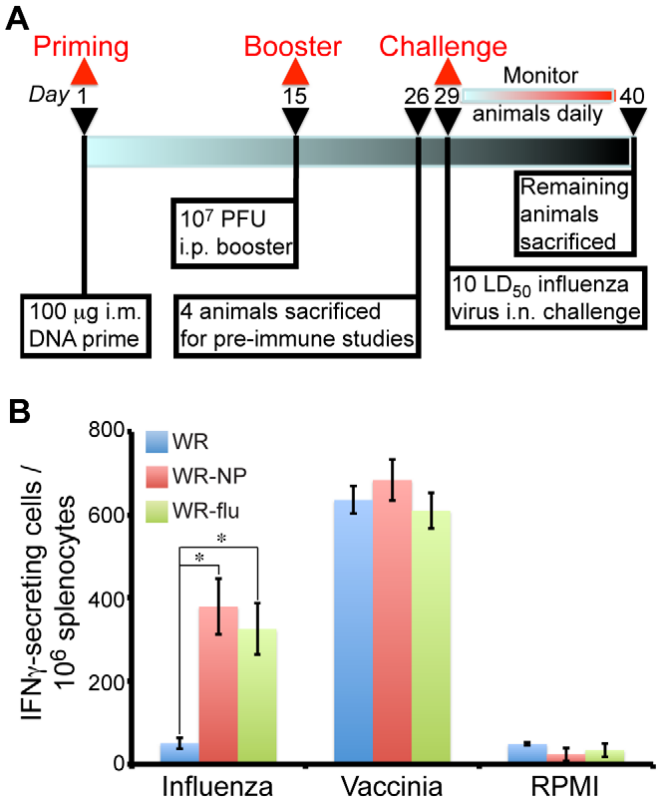
Immunogenicity of WR-NP and WR-flu in mice. (A) Immunization schedule. BALB/c mice were primed with 100 µg of DNA (either 100 µg pCIneo-NPmix or empty vector, or 50 µg pCIneo-NPmix+50 µg pCIneo-HANA) intramuscularly (i.m.) at the start of the vaccination protocol. Two weeks later, the mice were boosted by intraperitoneal (i.p.) infection with 10^7^ PFU of WR, WR-NP, or WR-flu. Eleven days post-boost, four mice were sacrificed to analyze the adaptive immune response. The remaining mice were challenged with influenza virus A/WSN/33, A/PR/8/34, or A/California/07/09. (B) Vaccine-elicited T cell responses of splenocytes 25 d after the start of the immunization protocol were measured in triplicate for each immunization group by fresh IFNγ ELISPOT assay following stimulation with influenza virus NP peptide TYQRTRALV, vaccinia virus E3 peptide VGPSNSPTF, or RPMI media alone. The results represent the mean number of IFNγ-secreting cells per 10^6^ splenocytes from three biological replicates ± standard deviations. *P* values from a two-tailed t test assuming nonequal variance are indicated (*, *P*<0.05).

We next examined the influenza virus-specific immune responses induced in mice by the WR-NP and WR-flu recombinant viral constructs. Four BALB/c mice for each group, WR, WR-NP, or WR-flu, were vaccinated according to the schedule in Figure 4A and sacrificed to evaluate the adaptive immune response elicited. Mice were primed with 100 µg DNA by intramuscular injection. 100 µg of empty DNA (pCIneo-Ø) was used for the WR group; 100 µg DNA containing the NPmix vector (pCIneo-NPmix) was used for the WR-NP group; 50 µg of NPmix DNA and 50 µg of DNA containing the NAHA vector (pCIneo-NAHA) was used for the WR-flu group. Two weeks post-prime, the animals were boosted by intraperitoneal infection of 10^7^ PFU of WR, WR-NP, or WR-flu. Eleven days post-boost, the adaptive immune response was evaluated using a fresh IFNγ ELISPOT assay with splenocyte stimulation using a peptide corresponding to influenza virus NP (TYQRTRALV) or vaccinia virus E3 (VGPSNSPTF).

As shown in Figure 4B, animals that were vaccinated with WR-NP or WR-flu exhibited a significant increase in splenic T cell responses against the NP peptide. Vaccination with WR-NP lead to a ∼7.6-fold increase in IFNγ-secreting cells while vaccination with WR-flu lead to a ∼6.5-fold increase, as compared to vaccination with WR alone. All vaccination protocols exhibited similar response levels to the E3 peptide, indicating that all viruses are replicating similarly in the vaccinated animals. We also performed experiments to determine the levels of neutralizing antibodies in the serum of vaccinated mice. However, we did not observe any significant increase in neutralizing antibodies against influenza virus nor against NA or HA in mice vaccinated with WR-NP or WR-flu versus WR alone (data not shown).

### Functional profile and polyfunctionality of WR-NP and WR-flu induced CD4^+^ and CD8^+^ T cell responses

To determine the phenotypic characteristics of the T cell populations activated after immunization with the DNA-prime/poxvirus-boost protocol, we utilized multiparameter intracellular flow cytometry staining (ICS) analysis to identify influenza virus-specific T cell responses. Splenocytes from four mice per group were cultured overnight and then stimulated with the NP-specific peptide in the presence of brefeldin for 6 h.

**Figure 5:**
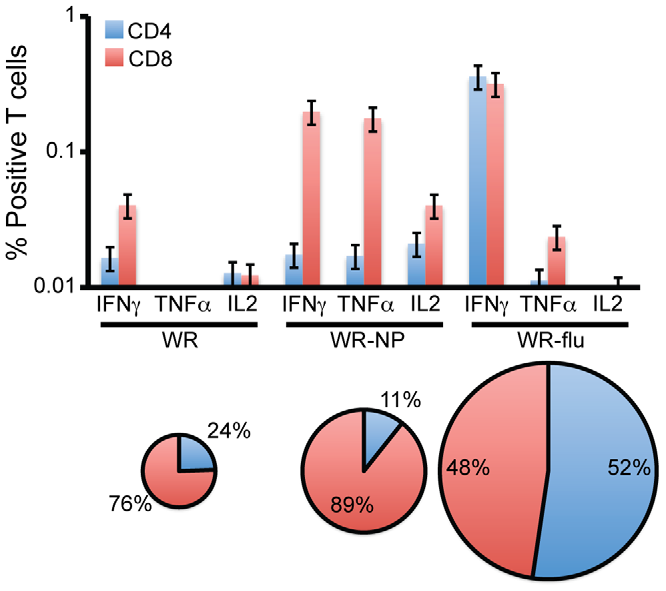
Phenotypic analysis of vaccine-induced CD4^+^ and CD8^+^ T cell responses. The same groups of splenocytes as described in Figure 4 were stimulated with the influenza virus NP-specific peptide and analyzed using polychromatic flow cytometry. The results represent the mean number of CD4^+^ and CD8^+^ T cells secreting IFNγ, TNFα, or IL2 in each immunization group using three biological replicates ± standard error. The background from unstimulated controls was subtracted in all cases. The pie charts represent the magnitude and percentage of CD4^+^ and CD8^+^ T cells secreting cytokines in each immunization group.

As shown in the pie charts of Figure 5, both the WR-NP and WR-flu vaccination protocols induced a greater magnitude of T cell responses as compared to WR alone. WR-NP induced a ∼2.9-fold increase in T cell response as compared to WR, and WR-flu induced a ∼9.1-fold increase. While T cells secreting cytokines following WR-NP vaccination were mostly CD8^+^, WR-flu induced both CD4^+^ and CD8^+^ T cells. However, the CD8^+^ T cells activated following WR-NP infection secreted both IFNγ and TNFα, while the T cells following WR-flu vaccination secreted mainly IFNγ.

**Figure 6:**
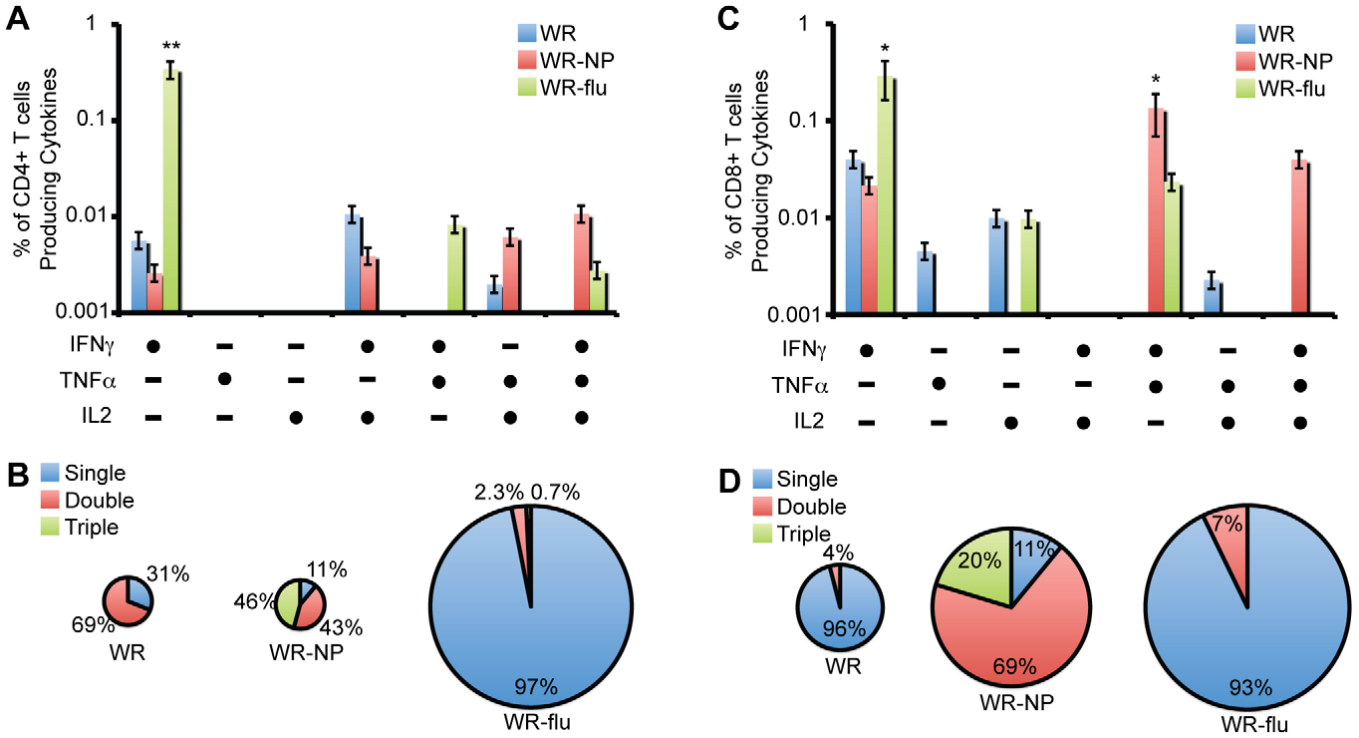
Polyfunctionality of influenza virus-specific CD4^+^ and CD8^+^ T cells. (A, C) Functional composition of CD4^+^ (A) or CD8^+^ (C) T cells responses against influenza virus NP peptide based on the secretion of IFNγ, IL-2, or TNFα. All the possible combinations of the responses are shown on the x-axis, whereas the percentages of the functionally distinct cell populations are shown on the y-axis. Bars correspond to the fraction of different functionally distinct T-cell populations within total CD4^+^ or CD8^+^ populations. Responses are grouped and color-coded on the basis of the number functions. (*, *P*<10^−5^; **, *P*<10^−25^) (B, D) The pie chart summarizes the data and each slice of the pie correspond to the fraction of CD4^+^ T cells with a given number of functions within the total CD4^+^ (B) or CD8^+^ (D) T cell populations. The size of the pie chart represents the magnitude of the specific influenza virus immune response induced.

The simultaneous measurements of three secreted cytokines allows for the assessment of the quality of the vaccine-induced CD4^+^ and CD8^+^ T cell responses. Upon analyzing concurrent secretion of IFNγ, TNFα, and IL2 by T cells, seven distinct influenza virus-specific CD4^+^ and CD8^+^ T cell populations can be identified. To further characterize the immunogenicity triggered in each immunized group, we assessed polyfunctional T cell responses. Regarding CD4^+^-secreting T cells (Figure 6A), we observed only a significant response following WR-flu vaccination, and this profile was not polyfunctional; CD4^+^ T cells secreted only IFNγ. However, WR-flu vaccination increased the overall magnitude of the CD4^+^ T cell response ∼19-fold (Figure 6B). With regard to CD8^+^ T cells, we did observe polyfunctionality following vaccination with WR-NP, but not WR-flu (Figure 6C). While vaccination with WR-flu elicited significantly higher levels of CD8^+^ T cells secreting IFNγ as compared to WR, vaccination with WR-NP exhibited significant levels of IFNγ/TNFα-secreting CD8^+^ T cells. While not statistically significant, WR-NP vaccination also lead to high levels of triple, IFNγ/TNFα/IL2-secreting, CD8^+^ T cells. As compared to vaccination with WR, the magnitude of the CD8^+^ T cell response was ∼3.5-fold higher for WR-NP and ∼5.7-fold higher for WR-flu (Figure 6D).

Overall, these results indicated that immunization with WR-NP induced a polyfunctional influenza virus-specific T cell response, while immunization with WR-flu was monofunctional with CD4^+^ and CD8^+^ T cells only producing IFNγ. Nevertheless, we show that WR-NP and WR-flu immunization improved the magnitude and quality of the anti-influenza virus response compared to WR. However, even though we observed an adaptive immune response characterized by polyfunctional T cells, we did not observe a memory response 53 days post- vaccination with the single NP peptide used as a test system (data not shown). This apparent lack of a memory response could be associated with the fact that we did not observe the production of neutralizing antibodies.

### Virus load is reduced in the lungs of mice upon challenge with influenza virus following vaccination

**Figure 7:**
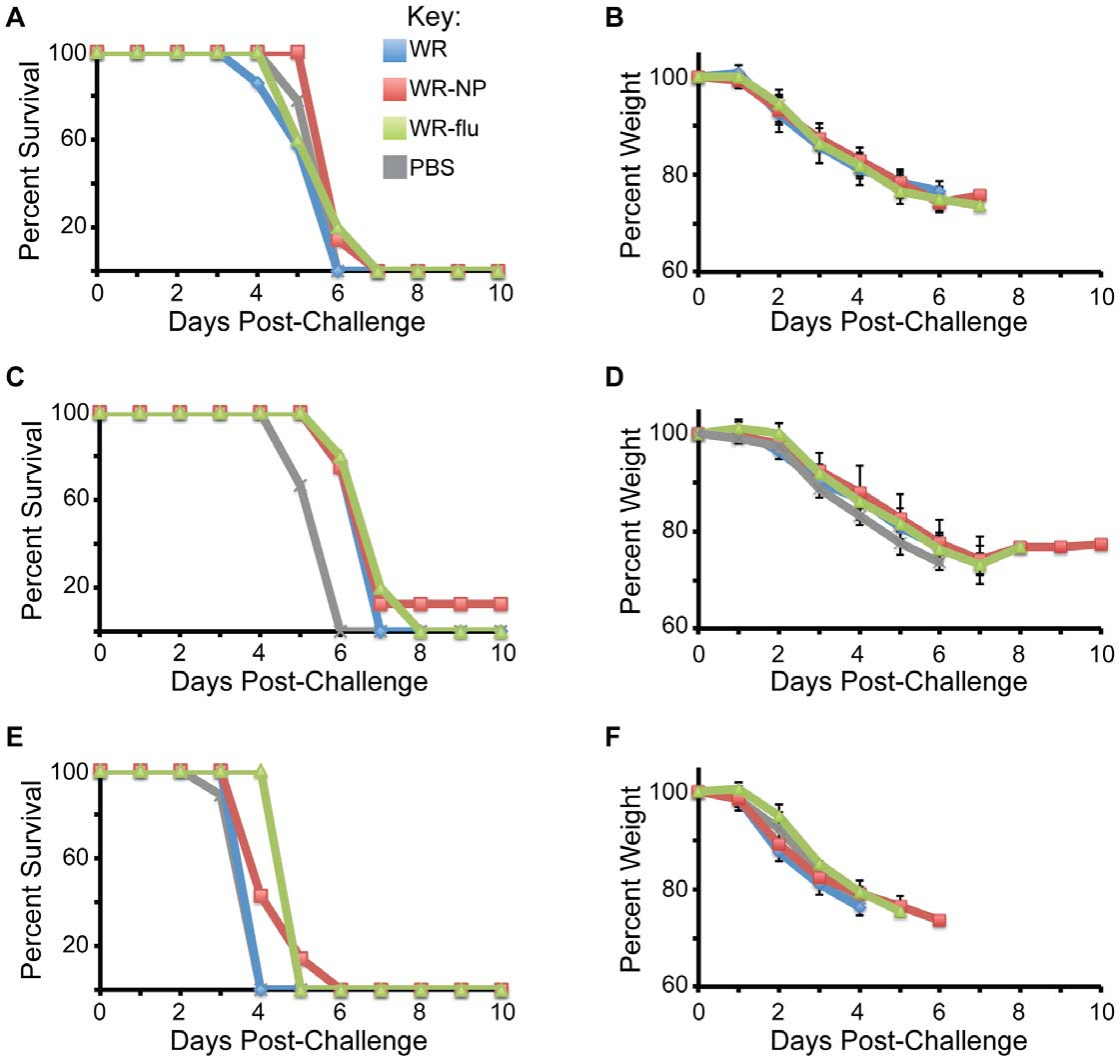
Vaccination delays mortality in influenza virus-challenged mice. Two weeks following the end of the vaccination protocol, 5–8 mice from each vaccination group and 9 mice from control PBS-inoculated animals were infected with 10×LD_50_ of the A/WSN/33 (A, B), A/PR/8/34 (C, D), or A/California/07/09 (E, F) strains of influenza virus. Mice were sacrificed when body weight reached 75% of starting weight.

**Figure 8:**
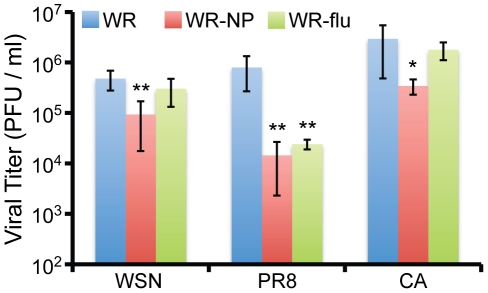
Vaccination reduces the levels of infectious virus in the lungs of influenza virus-challenged mice. Two weeks following the end of the vaccination protocol, mice from each vaccination group were infected with 10×LD_50_ of the A/WSN/33 (WSN), A/PR/8/34 (PR8), or A/California/07/09 (CA) strains of influenza virus. Mice were sacrificed at 5 d p.i. (except for some mice challenged with CA that died at 3–4 d p.i), and diaphragmatic lung lobes were isolated and homogenized. Levels of infectious virus were determined in triplicate by plaque assay on MDCK cells. The results represent the mean activity of 5–8 independent samples per group ± standard deviation. *P* values from a two-tailed t test assuming nonequal variance are indicated (*, *P*<0.05; **, *P*<0.01).

Upon measuring the adaptive immune response induced by vaccination with the recombinant DNA and vaccinia virus constructs, next we determined what effect vaccination had on challenge with influenza virus. Two weeks following the end of the vaccination protocol, mice from each vaccination group, along with 9 mice that had received PBS alone, were infected intranasally with 10×LD_50_ of the A/WSN/33 (WSN), A/PR/8/34 (PR8), or A/California/07/09 (CA) strains of influenza virus, corresponding to 10^4^, 10^3^, or 10^5^ PFU/mouse, respectively. Each day following challenge, mice were weighed and sacrificed when body weight reached at least 75% of their starting weight. While vaccination with WR-NP or WR-flu did not protect mice from death or weight loss, mortality was generally delayed by 1–2 days (Figure 7). However, we did observe a significant decrease in lung viral titers following vaccination with WR-NP and WR-flu as compared to WR (Figure 8). WR-NP vaccination decreased lung viral titers by ∼5.2-, ∼55-, and ∼8.6-fold upon challenge with WSN, PR8, and CA, respectively. Vaccination with WR-flu lead to a significant decrease in lung viral titer only upon challenge with PR8 (∼33-fold). We hypothesize that vaccination with WR-NP resulted in viral titers lower than vaccination with WR-flu because we observed a higher quality, polyfunctional T cell response during WR-NP vaccination.

Taken together, our study indicates that recombinant vaccinia viruses containing human T cell epitopes for influenza virus proteins embedded in NP or the conserved N-terminal of NA fused with the conserved C-terminal of HA can elicit specific T cell immune responses following heterologous vaccination utilizing a DNA-prime/poxvirus-boost protocol. While we observed significant levels of T cells secreting cytokines in a polyfucntional manner, this was not sufficient to protect mice from mortality; however, we did observe a decrease in lung viral replication upon vaccination with the recombinant viruses.

## Discussion

The vaccination strategy of heterologous prime-boost vaccination to elicit specific protective T cell responses has been widely established ever since its ground-breaking conception almost two decades ago [26], in which an influenza virus prime followed by vaccinia virus boost provided protection against *Plasmodium* infection in mice. Since this first evidence that vaccinia virus boost was required for strong activation of specific T cells, many different techniques have arisen, including the use of different viral vectors, DNA, and protein, in the presence and absence of adjuvant. In the present study, we constructed two different vaccinia virus vectors expressing recombinant influenza virus proteins, based on an NP backbone, which is one of the more well-conserved influenza virus proteins. Also utilized in our vaccine design were the N-terminal of NA and the C-terminal of HA, which are the well-conserved regions of H5N1 influenza virus subtypes. These recombinants were part of a DNA-prime/poxvirus-boost vaccination strategy in which a mammalian expression vector expressing the same sequences that were inserted into the vaccinia virus genome was used for the DNA priming stage. While our vaccination strategy elicited an immune response marked by CD4^+^ T cells expressing IFNγ, CD8^+^ T cells expressing IFNγ and TNFα, and resulted in decreased viral replication in the lungs of influenza-virus infected mice, these mice were not protected against mortality.

There has been some disparity regarding the ability of influenza virus NP in vaccine constructs to protect mice from mortality. A recent report describes a human vaccine trial using a vaccinia virus-based vaccine encoding the NP and M1 proteins against influenza virus [24]. This vaccine protocol elicited increased IFNγ-secreting CD8^+^ T cells in response to NP and M1. On the other hand, Lawson, et al., observed no protection following vaccination with vaccinia virus constructs encoding NP, even though they observed a reduction in lung viral load [20]. Ohba, et al. described a DNA-based vaccine based on the N-terminal of NP and observed protection upon challenge with influenza virus [18]. Saha, et al. also observed an improvement in vaccination using NP when used in conjunction with the VP22 gene of herpes simplex virus [17]. Finally, Altstein, et al. developed a vaccine using recombinant NP that included a proteolysis signal which provided some protection, especially when challenged with low doses of influenza virus [19]. Therefore, it was our goal to improve upon vaccine designs based on NP using a strategy in which we included human T cell epitopes for other influenza virus proteins within the NP backbone. A strategy based on multiple epitopes was successfully used to vaccinate against Japanese encephalitis virus in mice [27], as well as against hepatitis B virus and SIV [28], [29]. While we did not specifically test if every influenza virus epitope was correctly processed and presented, we know that the NP peptide used for the ELISPOT and ICS experiments was correctly presented. Following rationale design principles, we expect that the other epitopes would be presented [30]. However, future experiments with overlapping peptides for the entire influenza proteins to fully characterize T cell epitopes, perhaps using a transgenic humanized MHC class I mouse, should be performed. In addition to the multi-epitope NPmix recombinant vaccinia virus, we also constructed a recombinant virus encoding the conserved regions from HA and NA of H5N1 viruses to examine if the inclusion of more T cell epitopes would provide cross-clade protection when challenged with H1N1 viruses.

Although we observed that T cells were able to be stimulated to produce cytokines with influenza virus antigens, we did not observe a protection from lethality of the virus. A number of other studies also had difficulties in showing that HA and NP could provide protection from lethality [31], [32], [33], [34], [35], [36]. It may have been that our immunization scheme was sub-optimal, or most importantly, we used the mouse as a test animal. It should be highlighted that human T cell epitopes were used in the immunization regimen, and hence, these epitopes in the mouse might not be properly presented within MHC class I to stimulate T cells. This is why we had used only a known NP mouse epitope for stimulation of splenocytes for ELISPOT and ICS experiments. Also, it may be that the vaccinia virus boost suppressed the presentation of NP-generated T cell epitopes, as previously reported [37]. Since we challenged mice with 10×LD_50_, this may have been too high of a dose to observe the protective effects of vaccination. While we did observe a decrease in viral replication in the lungs of mice vaccinated with WR-NP and WR-flu, the amount of virus in the lungs was still greater than 10^4^ PFU/ml, which is high enough to cause lethality in mice [38]. It is likely that mouse lung pathology would be similar in all vaccination groups, and this would have contributed to mortality. Additionally, since our vaccine construct utilized a muti-epitope design, we may have observed increased immunogenicity upon stimulating splenocytes or T cells, if instead of the single NP peptide that we used pools of peptides spanning the influenza virus NP, M1, NS1, PB1, and PA proteins, similar to previously described studies [10], [11].

Many approaches exist to improve the vaccine design presented in this study, one of them being the use of adjuvants. Rapamycin has been shown to have immunostimulatory effects by improving antigen presentation and aiding in cytokine production from macrophages and dendritic cells. Furthermore, it is able to improve on the generation of memory CD8^+^ T cells following vaccination with a poxvirus vector [39], [40]. Recent results have shown that STING plays an important role in the generation of IFNγ-secreting CD8^+^ T cells [41]. Upon vaccination with a DNA vaccine, wild-type mice generated significantly increased amounts of IFNγ as compared to STING^−/−^ mice in response to peptide stimulation. These results suggest that the development of an adjuvant to stimulate STING during vaccination would augment the immune response to antigen presentation. In addition to the use of adjuvants, many other future directions exist for the improvement of our vaccine design to move closer to a universal influenza virus vaccine. Our constructs that elicit T cell responses could be combined with a vaccine specifically design to elicit a humoral B cell-producing neutralizing antibody response. Secondly, we would have to test the vaccine for its efficacy in protection after challenge with other influenza virus subtypes, such as H5N1, H3N2, H9N2, and H7N7, all of which contain conserved NP genes. It would also be prudent to challenge with influenza B subtypes, since seasonal influenza virus vaccines contain an attenuated B subtype virus. Since challenge with wild-type H5N1 strain of influenza virus requires high biosafety levels, we could challenge with a recombinant strain of PR8 that expresses HA and NA from H5N1 viruses.

In summary, our study describes the design and development of recombinant DNA and vaccinia virus vaccine constructs for use in a DNA-prime/poxvirus-boost vaccine protocol as proof-of concept protocol for a universal vaccine candidate against influenza virus. Since these experiments were performed with the aim to show proof-of-principle, we used the replication-competent WR strain of vaccinia virus; any future vaccine trials, especially in humans, non-replicative and safe, attenuated vaccinia virus strains, such as MVA or NYVAC [12] must be used. Our construct contains multiple T cell epitopes against influenza virus antigens, which were aimed to broaden and increase immune responses upon influenza virus challenge. While our construct elicited an immune response marked by increased CD4^+^ and CD8^+^ T cells expressing IFNγ or TNFα, and resulted in decreased viral replication in the lungs of influenza-virus infected mice, these mice were not protected against mortality. Taken together, our results suggest that a human mutli-epitope vaccine design in a DNA prime/poxvirus boost approach can stimulate the breath and quality of specific T cell immune responses to influenza virus antigens leading to reduction in viral load in the lungs. This reduction might play an important role to limit influenza virus replication in a natural infection and to develop host immune resistance. The protocol of immunization described here can be further improved through the use of a recombinant attenuated vaccinia virus strain, like MVA, and combination with adjuvants and vectors inducing neutralizing antibodies to influenza virus proteins. Hence, a vaccine construct that elicits broad T cell responses and limits virus replication to some extent, as described here, could be combined with a second vaccine that elicits a neutralizing antibody response to further restrict the virus load. Together, such a vaccine strategy could bring us closer to creating a universal vaccine against influenza virus infection.

## Materials and Methods

### Cells, viruses, and infections

BSC40 monkey kidney epithelial cells and Madin-Darby canine kidney (MDCK) cells (ATCC) were grown as monolayers in supplemented high glucose Dulbecco's modified Eagle's medium (hgDMEM) supplemented to contain 2 mM L-glutamine, 0.1 mM nonessential amino acids, Fungizone Amphotencin B (0.5 µg/ml), penicillin G (100 units/ml), streptomycin sulfate (100 µg/ml) and 10% newborn calf serum (NCS) or fetal calf serum (FCS) (Sigma), respectively. Wild-type vaccinia virus (strain WR) and recombinant WR viruses expressing influenza virus proteins were grown and plaque-purified on monkey BSC-40 cells, purified by two 45% (w/v) sucrose cushions, and titrated on BSC40 cells by plaque assay.

Near-confluent monolayers of cells were mock-infected or infected with vaccinia virus diluted in supplemented hgDMEM to the indicated multiplicity of infection (MOI). After 1 h of adsorption at 37°C, virus and medium was removed. Fresh supplemented hgDMEM containing 2% NCS was added to the cells and infections were allowed to proceed at 37°C until the indicated time post-infection.

### Mouse inoculations

At day 1, six- to eight-week old mice BALB/c mice (Harlan) were anesthetized with isoflurane and injected intramuscularly with PBS alone or 100 µg of empty pCIneo vector or containing NPmix, NA-HA, or both (n = 19–27). At day 15, mice were anesthetized and infected intraperitoneally with 10^7^ PFU of WR, WR-NP, or WR-flu. At day 26, four animals were sacrificed for adaptive immune response analysis. At day 29, the remaining mice were anesthetized and challenged intranasally with 10×LD_50_ of the A/WSN/33, A/PR/8/34, or A/California/07/09 strains of influenza virus (n = 5–8 for each group). Animals were weighed each day for ten days and sacrificed when they lost at least 25% of their starting body weight. Blood and lung tissue was collected from each mouse at the time of sacrifice. All experiments were performed in a specially separated negative-pressure HEPA (high-efficiency particulate air)-filtered biosafety level 2 laboratory. All animals were handled in strict accordance with good animal practice as defined by the relevant national, international, and/or local animal welfare bodies, and with the Royal Decree (RD 1201/2005). All animal work was approved by the Ethical Committee of Animal Experimentation (CEEA-CNB) of the Centro Nacional de Biotecnología (CNB-CSIC). Permit number: 10015.

### Generation and verification of recombinant vaccinia viruses

Genes for expression of the recombinant influenza virus proteins, NPmix and NA-HA (Figure 1B) were designed by us and sent to GeneArt® for synthesis and insertion into the pBlueScript vector. During the optimization process for the sequences, the following cis-acting sequence motifs were avoided: internal TATA-boxes, chi-sites and ribosomal entry sites; AT-rich or GC-rich sequence stretches; ARE, INS, CRS sequence elements; repeat sequences and RNA secondary structures; (cryptic) splice donor and acceptor sites, branch points; and AscI, FseI, NotI, PmlI and SalI sites. The influenza virus genes were codon optimized and contain either an N-terminal FLAG tag (NPmix) or a C-terminal His tag (NA-HA). The genes were individually cloned into pCIneo for mammalian expression, and also cloned into the TK locus of vaccinia virus using the transfer vector pCyA. In this vector NPmix was inserted alone, or in front of NA-HA, both of which were driven by the vaccinia virus early/late promoter (pE/L). Homologous recombination in the wild-type vaccinia virus strain WR was performed as previously described [42]. Recombinant virus containing NPmix was called “WR-NP,” and virus containing both NPmix and NA-HA was called “WR-flu”. Expression of the influenza genes was driven by a synthetic early/late virus promoter (Figure 1A).

### Protein analyses and plaque assays

Following vaccinia virus infection or transfection with the pCIneo expression vectors using Lipofectamine 2000 (Invitrogen) following the manufacturer's instructions, cells were lysed at the indicated times p.i. in disruption buffer (0.5% Triton X-100, 50 mM KCl, 50 mM NaCl, 20 mM Tris-HCl [pH 7.5], 1 mM EDTA, 10% glycerol, 1× Complete protease inhibitor (Roche), 25 mM β-glycerophosphate, 1 mM Na_3_VO_4_). Total protein content was determined for clarified cell lysates by using the BCA protein assay kit (Pierce). Lysates were separated by SDS-PAGE with the same amount of total protein being loaded into each lane and then transferred onto nitrocellulose membranes. Immunoblots were blocked for 1 h in PBS containing 0.5% Tween 20 and 5% nonfat dry milk, washed in PBS containing 0.05% Tween 20, and incubated at 4°C overnight with a mouse monoclonal actin antibody (MP Biochemicals), a rabbit polyclonal NP antibody (a kind gift from Adolfo García-Sastre), or a sheep polyclonal HA antibody (provided by the National Institute for Biological Standards and Control) in PBS containing 0.5% Tween 20 and 1% nonfat dry milk. Subsequently, membranes were washed and incubated for 2 h with horseradish peroxidase-conjugated goat anti-mouse, goat anti-rabbit, or donkey anti-sheep immunoglobulin G (Sigma), and bound antibodies were detected with Amersham ECL Western blotting detection reagent (GE Healthcare).

At the indicated times post-infection, vaccinia virus-infected cells and cell media supernatant were collected and assayed in triplicate for viral yield by standard plaque assay on BSC40 cells. For influenza virus-infected mice, diaphragmatic lung lobes from each animal were weighed, homogenized in PBS, and samples were then assayed in triplicate for viral yield by standard plaque assay on MDCK cells. Viral yields were calculated according to the formula: log yield_t = x_ = [log_10_(PFU/ml)_t = x_]/[log_10_(PFU/ml)_t = 0_], where *t* is time and *x* is the time post-infection.

For immunostaining of vaccinia virus plaques, infected cells were fixed 24 h p.i. with 1∶1 methanol∶acetone, washed in PBS, then incubated for 2 h with primary antibodies for vaccinia virus (WR strain) or influenza virus NP diluted in PBS containing 3% FCS. Cells were then washed and incubated for 1 h with horseradish peroxidase-conjugated goat anti-rabbit diluted in PBS containing 3% FCS. The spots were developed in PBS containing 1 mg/ml of the substrate 3,3′-diaminobenzidine tetrahydrochloride (Sigma) with 0.03% hydrogen peroxide and 0.03% nickel sulfate.

### Immunofluorescence

Following influenza virus infection of cells cultured on glass coverslips, cells were fixed in 2% paraformaldehyde in PBS, permeabilized in 0.1% Triton X in PBS, washed with 2.5% FCS and 10 mM glycine in PBS, and then blocked with 10% FCS in PBS. Cells were then incubated for 2 h with primary antibodies recognizing influenza virus NP, HA, or vaccinia virus 14 K (A27 gene) diluted in 10% FCS in PBS. Subsequently, cells were washed and incubated for 1 h with Alexa 488- or 586-conjugated anti-rabbit immunoglobulin G (IgG) (Invitrogen), or fluorescein isothiocyanate (FITC)-conjugated donkey anti-sheep IgG (Jackson Immunoresearch). Cells were washed and incubated for 20 min with DAPI (4′,6′-diamidino-2-phenylindole) (Sigma) and mounted onto glass slides using ProLong Antifade reagent (Invitrogen). Cells were imaged with the Leica TCS SP5 multispectral confocal microscope (Leica Microsystems) using photomultipliers for laser lines 405, 488, and 561 nm. LAS AF v.2.3.6 software was used for image acquisition.

### IFNγ ELISPOT assay

The vaccine-specific cellular immune response in mice was determined using ELISPOT assay measuring the secretion of IFNγ by splenocytes after stimulation with a peptide specific for influenza virus NP (TYQRTRALV), as previously described [10], [11]. Briefly, eleven days after boosting with vaccinia virus, mice were sacrificed and splenocytes depleted of red blood cells were isolated. 10^6^ splenocytes were plated in triplicate in 96-well nitrocellulose-bottomed plates previously coated with 6 mg/ml of anti-mouse IFNγ mAb R4-6A2 (Pharmingen). Cells were stimulated with the influenza virus-specific peptide (2 µg/ml), a positive control peptide against the E3 protein of vaccinia virus (VGPSNSPTF, 5 µg/ml), or without peptide as a negative control. 48 h after stimulation, cells were washed and those secreting IFNγ were developed using a biotin-streptavidin sandwich system and counted using a stereomicroscope.

### Intracellular Cytokine Staining (ICS) assay

Multiparameter flow cytometry was performed as previously described [10], [11]. Briefly, 10^6^ splenocytes were stimulated with the peptides described above in the presence of 1 µl/ml Brefeldin (BD Bioscience) for 6 hours in a 96-well plate. The cells were then washed, stained with the LIVE/DEAD Kit (Invitrogen), and Fc receptors were blocked using CD16/CD32 antibodies (BD Biosciences). The cells were then stained with the surface-specific mouse antibodies, CD4-Alexa700, CD3-FITC, and CD8-PerCP (BD Biosciences). Cells were permeabilized using the BD Cytofix/Cytoperm Kit and were stained for the intracellular cytokines, IFNγ-APC, IL2-PE and TNFα-PECy7. Sample acquisition was performed with an LSRII Flow Cytometer and FACSDiva software (BD Biosciences) and was further analyzed with FlowJo (Tree Star). All statistical analysis was performed as previously described [10], [11].

## Supporting Information

Figure S1 **Alignment of nucleoprotein (NP) from various influenza virus subtypes.** The NP protein of five influenza virus strains of different subtypes (H1N1, H2N3, H5N1, H9N2, and H7N7, from top to bottom) was aligned. The conserved region, as indicated by red underline, from amino acids 19–498, was used as the backbone for the NPmix multi-epitope protein.
(TIF)

Figure S2 **Alignment of hemagglutinin (HA) and neuraminidase (NA) from different strains of the H5N1 influenza virus subtype.** The NA protein (A) or HA protein (B) from seven or eight different H5N1 viruses were aligned. The conserved N-terminal of NA (amino acids 107–231) and conserved C-terminal of HA (amino acids 347–511), as indicated by red underline, were fused and used for the NA-HA construct along with NPmix in the WR-flu recombinant virus.
(TIF)
