# Supplementary figures and images for: A Human Multi-Epitope Recombinant Vaccinia Virus as a Universal T Cell Vaccine Candidate against Influenza Virus
Source: PLoS One. 2011 Oct 5;6(10):e25938. doi: 10.1371/journal.pone.0025938 (PMC3187825; doi:10.1371/journal.pone.0025938)

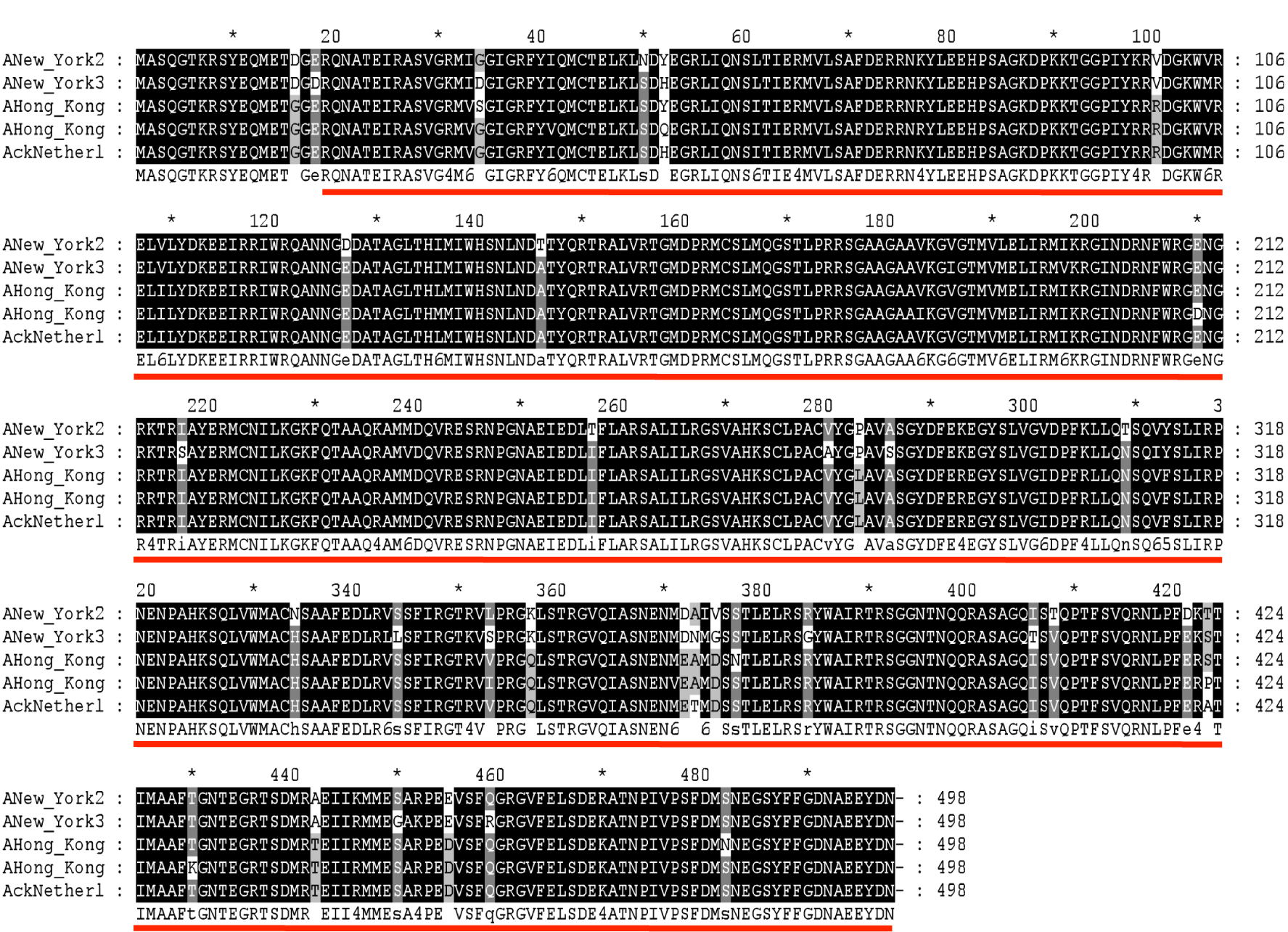

Supplement: Figure S1 — Alignment of nucleoprotein (NP) from various influenza virus subtypes. The NP protein of five influenza virus strains of different subtypes (H1N1, H2N3, H5N1, H9N2, and H7N7, from top to bottom) was aligned. The conserved region, as indicated by red underline, from amino acids 19–498, was used as the backbone for the NPmix multi-epitope protein. (TIF) [file pone.0025938.s001.tif]

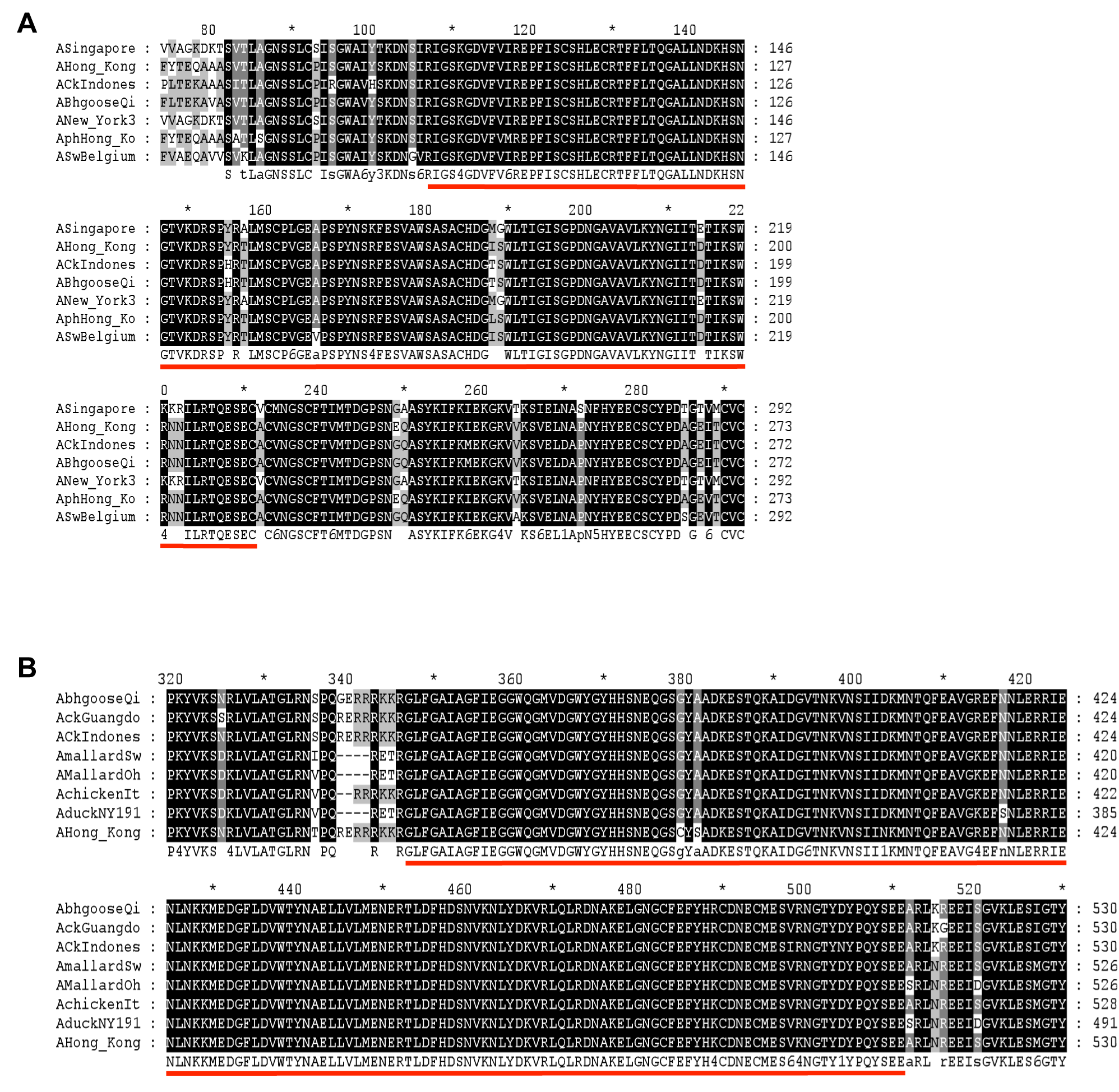

Supplement: Figure S2 — Alignment of hemagglutinin (HA) and neuraminidase (NA) from different strains of the H5N1 influenza virus subtype. The NA protein (A) or HA protein (B) from seven or eight different H5N1 viruses were aligned. The conserved N-terminal of NA (amino acids 107–231) and conserved C-terminal of HA (amino acids 347–511), as indicated by red underline, were fused and used for the NA-HA construct along with NPmix in the WR-flu recombinant virus. (TIF) [file pone.0025938.s002.tif]
